# Intelligence

**Published:** 1831-06

**Authors:** 


### INTELLIGENCE.

---

### LONDON UNIVERSITY.

Dr. Elliotson is appointed to the chair of Medicine in the University of London. It is reported that Mr. Quain is a candidate for the chair of Anatomy.

---

### LONDON MEDICAL SOCIETY.

*Expulsion of* Dr. Ramadge. In consequence of Dr. Ramadge having published a letter in defence of the very well known St. John Long, the members of this Society held a special meeting lately, at which it was determined, by a majority of twenty-three, that Dr. Ramadge should be expelled from the Society. The numbers were, for his expulsion, twenty-four; against it, one! We trust Mr. Long will not forget the zeal of his advocate: he deserves reward for the boldness he has manifested in coming forward in *such* a cause.

---

### GENERAL DISPENSARY, 36, ALDERSGATE STREET.

*A Course of Practical Chemistry.* By Jonathan Pereira, F.L.S. &c.

This Course will consist of a series of experiments and operations, to be performed by the students themselves, in order to give them that dexterity and readiness in the performance of chemical experiments, which can only be acquired by practice. Mr. Pereira will superintend each step of the process, explain any difficulties that may occur, and point out whatever may be interesting.

The experiments will be arranged so as to form a Course of Chemistry, and will, therefore, include the preparation of the gases, and demonstration of their properties; the chemical properties of the metals, and their principal combinations; and of the vegetable and animal bodies. Among other points to which the attention of the student will be especially directed, may be mentioned the chemical processes of the Pharmacopœia; the methods of estimating the specific gravities of solids, liquids, and gases; the construction of thermometers; the use of the blowpipe; the formation of test tubes; the analysis of substances supposed to be poisonous; the examination of urinary calculi; the theory of equivalents; the construction of diagrams, &c.

By these means, it is presumed that the pupil may easily acquire a good practical knowledge of the most important chemical facts. In the university of Edinburgh, similar courses of practical chemistry, under the superintendence of Dr. Boswell Reid, have been found eminently successful. The class will meet twice a week, and the course will occupy about three months.

### LITERARY NOTICE.

In the press, A Manual of State and Forensic Medicine, compiled from the best Medical and Legal Works: comprising, 1. The Ethics of the Medical Profession, ancient and modern; Moral Statutes of the British Universities and Colleges; 2. The Charters and Statutes relating to Physicians, Surgeons, Apothecaries, Obstetricians, Chemists, Druggists, and Empirics in the British Dominions; 3. The Rights, Privileges, and Immunities of the Faculty; 4. The Civil and Criminal Cases in which Medical Evidence is required; 5. All Medico-legal Questions, with the latest Decisions: being an Analysis of a Course of Lectures on Medical Jurisprudence, annually delivered in London, and intended as a Compendium for the use of Barristers, Solicitors, Magistrates, Coroners, and Medical Practitioners. By MICHAEL RYAN, M.D. &c.

### METEOROLOGICAL JOURNAL,

By Messrs. HARRIS and Co. Mathematical Instrument Makers, 50, High Holborn.

| | Moon. | Rain gauge. | Thermom. | | | Barometer. | | De Luc's Hygrometer. | | Winds. | | Atmospheric Variation. | | |
|---|---|---|---|---|---|---|---|---|---|---|---|---|---|---|
| Apr. | | | 9 a.m. | max. | min. | 9 a.m. | 10 p.m. | 9 a.m. | 10p.m. | 9 a.m. | 10p.m. | 9 a. m. | 2 p.m. | 10 p.m. |
| 20 | | | 49 | 57 | 42 | 29.63 | 29.53 | 58 | 58 | NNE | NNE | Fine | Fine | Fine |
| 21 | | | 47 | 56 | 46 | .32 | .30 | 59 | 61 | NE | ESE | Cloudy | Cloudy | — |
| 22 | | | 52 | 54 | 49 | .26 | .34 | 64 | 62 | ESE | E | Cloudy | Fine | — |
| 23 | | | 55 | 61 | 48 | .34 | .46 | 62 | 61 | NNE | E | Cloudy | Cloudy | Cloudy |
| 24 | | | 53 | 57 | 51 | .58 | .75 | 61 | 60 | NW | NW | Fine | Fine | — |
| 25 | | | 57 | 61 | 45 | .83 | .81 | 60 | 58 | NNW | WSW | Cloudy | — | Fine |
| 26 | ○ | | 48 | 55 | 50 | .64 | .61 | 58 | 57 | S | W | Fine | — | — |
| 27 | | | 54 | 60 | 48 | .45 | .31 | 56 | 56 | SSE | S | Foggy | — | Cloudy |
| 28 | | | 53 | 58 | 46 | .16 | .15 | 57 | 60 | S | S | Cloudy | Cloudy | — |
| 29 | | | 52 | 59 | 50 | .05 | .05 | 62 | 60 | SSE | S | Sho'ry | — | Rain |
| 30 | | .65 | 60 | 60 | 47 | .04 | .10 | 59 | 55 | SW | NNW | Fine | Fine | Sho'ry |
| May | | | | | | | | | | | | | | |
| 1 | | | 53 | 59 | 48 | .16 | .31 | 60 | 58 | W | W | — | Rain | Cloudy |
| 2 | | .18 | 58 | 61 | 50 | .36 | .44 | 57 | 61 | SE | E | — | — | Fine |
| 3 | | | 57 | 64 | 51 | .48 | .50 | 64 | 61 | SW | WSW | Sho'ry | Cloudy | Cloudy |
| 4 | | .30 | 60 | 61 | 46 | .38 | .39 | 58 | 57 | S | WSW | Overc. | Fine | Fine |
| 5 | ☾ | | 53 | 54 | 42 | .34 | .41 | 57 | 56 | W | WSW | Fine | Rain | — |
| 6 | | .22 | 50 | 50 | 36 | .50 | .71 | 54 | 50 | NW*v*. | NE | — | Fine | — |
| 7 | | | 48 | 52 | 38 | .84 | .97 | 49 | 48 | NW | ESE | — | — | — |
| 8 | | | 52 | 59 | 42 | 30.01 | 30.11 | 48 | 49 | E | E | — | — | — |
| 9 | | | 53 | 57 | 40 | .14 | .07 | 49 | 50 | NE | E | — | — | — |
| 10 | | | 49 | 58 | 48 | .04 | .01 | 51 | 51 | E | E | Sleet | — | — |
| 11 | ● | | 57 | 60 | 41 | .02 | .02 | 51 | 50 | SE | E | Fine | — | — |
| 12 | | | 56 | 62 | 46 | 29.94 | 29.84 | 48 | 48 | E | E | — | — | — |
| 13 | | | 54 | 59 | 43 | .79 | .86 | 48 | 47 | NE | ESE | — | — | — |
| 14 | | | 52 | 59 | 45 | .92 | .90 | 46 | 45 | ENE | SE | — | — | — |
| 15 | | | 54 | 60 | 42 | .90 | .91 | 45 | 45 | S | S | — | — | — |
| 16 | | | 50 | 61 | 50 | .93 | .95 | 45 | 45 | N | NNE | — | — | — |
| 17 | | | 63 | 65 | 52 | 30.01 | .93 | 46 | 45 | SE | ESE | — | — | — |
| 18 | ☽ | | 65 | 65 | 54 | 29.79 | .73 | 45 | 43 | E | E *va*. | — | — | — |
| 19 | | | 63 | 65 | 55 | .61 | .57 | 43 | 45 | E *va*. | SE | — | — | Rain |

The quantity of Rain fallen, in the month of April, was 1 inch.

